# Impact of klotho on the expression of SRGAP2a in podocytes in diabetic nephropathy

**DOI:** 10.1186/s12882-022-02765-z

**Published:** 2022-04-18

**Authors:** Donghua Jin, Miao Jia, Yuxian Xie, Lihua Lin, Hong Qiu, Guoyuan Lu

**Affiliations:** 1 Department of Nephrology, People’s Hospital of Suzhou New District, Suzhou, 215129 China; 2 Department of Nephrology, The First Affiliated Hospital of Soochow University, Jiangsu province, Suzhou, China

**Keywords:** Diabetic nephropathy, Podocyte, Klotho, SRGAP2a

## Abstract

**Background:**

Diabetic nephropathy (DN) is the major cause of kidney failure, and glomerular podocytes play critical roles in the pathogenesis of DN by maintaining the glomerular structure and filtration barrier. Klotho and Slit-Robo GTP activating protein 2a (SRGAP2a) have been indicated to play protective roles in reducing kidney injury, but whether there is an internal relationship between these two factors is unclear.

**Methods:**

In this study, we cultured differentiated rat podocytes in vitro and measured the SRGAP2a expressions by immunofluorescence staining, quantitative real-time PCR (qRT-PCR) and western blotting, after siRNA-mediated transforming growth factor β1 (TGF-β1) silencing, TGF-β1 overexpression and in the presence of a reactive oxygen species (ROS) inhibitor. And we detected the expressions of SRGAP2a, small mother against decapentaplegic (Smad)2/3, phosphorylated-Smad2/3 (p-Smad2/3), Smad7, and NAD(P)H oxidase 4 (NOX4), ROS levels and podocyte cytoskeletal remodelling under high glucose (HG) and exogenous klotho conditions. In addition, we performed haematoxylin–eosin (HE) staining and immunohistochemistry with diabetic rat models to confirm the in vitro results.

**Results:**

The results indicated that SRGAP2a expression was significantly upregulated under siRNA-mediated TGF-β1 silencing conditions or after adding a ROS inhibitor, but significantly downregulated with TGF-β1 overexpression, in the presence of HG. The supplementation of exogenous klotho under HG conditions significantly increased the SRGAP2a expression, remodelled the actin cytoskeleton and altered the expressions of Smad2/3, p-Smad2/3, Smad7 and NOX4 and reduced the ROS generation in podocytes. Moreover, klotho administration protected kidney injury in DN rats.

**Conclusions:**

This study indicated that klotho may modulate the expression of SRGAP2a by regulating the ROS and TGF-β1 signalling pathways and provided theoretical support for klotho protein as a novel therapeutic strategy for treating DN patients.

**Supplementary Information:**

The online version contains supplementary material available at 10.1186/s12882-022-02765-z.

## Background

Diabetic nephropathy (DN) is the leading cause of chronic kidney disease (CKD), which has been recognized as a public health disease worldwide because it creates a high risk of cardiovascular disease and other complications [1–4]. Accumulating evidence has shown that glomerular podocytes, which are glomerular visceral epithelial cells residing on the urinary side of the glomerular basement membrane (GBM), play a critical role in the pathogenesis of DN by maintaining the glomerular structure and filtration barrier [5–8].

Klotho was initially identified as an anti-ageing gene [9–12]. There are three members in this family: α-, β-, and γ-klotho [13]. In this paper, the term klotho in the subsequent text refers to α-klotho. Klotho is a single transmembrane protein that consists of a small intracellular domain in the C terminus and a large extracellular domain in the N terminus [12, 14]. Membrane-bound klotho is suggested to be a coreceptor mainly for FGF23 signalling, regulating kidney phosphate excretion [15–17]. The extracellular domain can be cleaved by a disintegrin and metalloproteinase (ADAM)-10 and ADAM-17 at the α cut site to become soluble klotho, which contains two functional repeats named Kl1 and Kl2 and are involved in the circulation [12, 18, 19]. Generally, soluble klotho, which refers to this degradation fragment of membrane-bound klotho, is the functional form and is widely present in the blood, urine and cerebrospinal fluid [20–22]. Soluble klotho has been suggested to be an ideal biomarker for CKD based on findings from clinical and animal studies that klotho deficiency seems to be significantly associated with podocyte injury and kidney fibrosis in human kidneys [23]. Moreover, extracellular supplementation is considered a novel therapeutic strategy for CKD to restore klotho levels and/or promote endogenous expression [24, 25]. Previous studies indicated that soluble klotho could inhibit transforming growth factor β1 (TGF-β1) activity to reduce kidney fibrosis by directly binding to the TGF-β1 receptor and preventing TGF-β1 signalling [24, 26].

The normal function of podocytes depends on their actin cytoskeleton. Previous studies revealed that Rho family GTPases regulate podocyte actin dynamics, and abnormalities in Rho GTPase activity may result in podocyte mobility and induce proteinuria [7, 27–30]. Slit-Robo GTP activating protein 2a (SRGAP2a), a member of the Rho GTPase large family, has been shown to be primarily enriched in podocytes and is tightly correlated with the estimated glomerular filtration rate (eGFR) and proteinuria of DN patients [7]. The expression of SRGAP2a was shown to be decreased in DN patients and *db/db* mice, while increasing SRGAP2a levels in podocytes in *db/db* mice efficiently rescued the DN state [7]. Recent studies revealed that the expression of SRGAP2a decreased significantly in the presence of high glucose (HG) or TGF-β1, and exogenous SRGAP2a largely rescued podocytes from HG- and TGF-β1-induced damage [7, 31]. SRGAP2a has been shown to play protective roles against podocyte injury and proteinuria in DN [7, 31]. In addition, ROS production was a key player progressing diabetic kidney diseases and was closely associated with TGF-β1 signaling [32, 33].

Therefore, there is a close relationship between the levels of klotho, SRGAP2a, TGF-β1 and ROS signaling in CKD, including DN. However, whether klotho attenuates kidney injury by regulating SRGAP2a in podocytes remains unclear. In this study, we cultured differentiated rat podocytes and measured the expression of SRGAP2a, the reactive oxygen species (ROS) levels, and key factors in TGF-β1 and ROS signalling with or without exogenous klotho under HG conditions and analysed the relationship between klotho and SRGAP2a. And we confirmed the in vitro findings with diabetic rat models. The results provide theoretical support for klotho protein as a novel therapeutic strategy for treating DN patients.

## Materials and methods

### Primary podocyte culture

We isolated two rat kidneys under aseptic conditions and dissected and gently ground the kidney cortex. The tissues were rinsed by sequential passage through 100 μm and 200 μm sieves at 4 °C. Glomeruli were collected on a 200 μm sieves, and 2 g/L type IV collagenase (Sigma, china) was properly digested at 37 °C for about 15 min. Under the observation with the inverted microscope, when a few cells were dissociated in the medium, added completed Dulbecco's modified Eagle's medium (DMEM) (Hyclone, USA) to terminate the digestion. Centrifuge and wash twice at 1000 r/min for 10 min, and resuspended in DMEM, inoculated into three 75 cm^2^ culture flasks lined with rat tail collagen and incubated at 37 °C and 5% CO_2_ for 7–8 days. The majority of cells observed with an electron microscope (Leica TCS SP8 STED, Germany) were primary podocytes.

### Immunofluorescence staining

Primary podocytes were cultured for another 7 days after being trypsinized and subsequently passaged. Podocyte morphology was observed with an inverted phase contrast microscope (Leica TCS SP8 STED, Germany) when the cells covered the bottom of the dish. The cells were fixed with cold acetone and processed for immunofluorescence staining for the podocyte markers nephrin and synaptopodin.

### Cell transfection

All siRNAs used in this study were synthesized by GenePharma (Suzhou). The siRNA-TGF-β1 (TGF-β1 siRNA-2) sequence consisted of a 21-nucleotide sense strand (5'-GACAAGUUCAAGCAGAGUACA-3') and an antisense strand (5'-UACUCUGCUUGAACU UGUCAU-3'). Podocytes were inoculated in 24-well plates and transfected with the designed siRNAs to mediate TGF-β1 silencing with Lipofectamine™ 2000 transfection reagent according to the manufacturer’s instructions (Thermo Fisher, US). Twenty-four hours after transfection, the podocytes were treated with HG and harvested for subsequent analysis such as SRGAP2a immunofluorescence staining, qRT-PCR and western blotting.

## Quantitative real-time PCR (qRT-PCR)

Podocyte samples were treated with mannitol (100 mM), glucose (100 mM), glucose (100 mM) plus acetylcysteine (10 μM) and glucose (100 mM) plus klotho (2000 pM) for 24 h, and samples without additional sugar treatment were used as negative controls. Then, the medium was removed from the 6-well cell culture plate, and 1 × PBS solution (Sangon Shanghai) was added to wash the cells gently. Next, the 6-well plate was placed on ice, and 800 μl of TRIzol reagent (Sigma, US) was added to each sample, which were repeatedly pipetted to dislodge all adherent cells, and the cells were transferred to 1.5 ml EP tubes. The total RNA of each sample was extracted according to the manufacturer’s instructions and reverse transcribed with the PrimeScript™ RT reagent kit (TaKaRa, Japan). qRT-PCR was performed with SYBR Green detection mix (TaKaRa, Japan). The relative expression levels of genes in this study were normalized to actin expression, analysed by the 2^−ΔΔCt^ method, and summarized from three separately harvested podocyte samples. The primers used for qRT-PCR were showed in Supplemental Table 1.

### Western blot analysis

Podocyte samples were treated as described above for qRT-PCR. Total protein was extracted from each sample to prepare cell lysates using RIPA Lysis Buffer (Sangon, Shanghai, China) and stored at -20 °C. The bicinchoninic acid (BCA) protein quantification method was used to ensure that the concentration of each sample was basically equal. Protein samples were subjected to sodium dodecyl sulfate–polyacrylamide gel electrophoresis (SDS-PAGE) and transferred to PVDF membranes with electrophoresis systems (Tanon VE180 and Tanon VE186, Shanghai, China). The PVDF membranes were blocked with 5% (w/v) skimmed milk powder for 2 h and incubated at 4 °C overnight with the following primary antibodies: rabbit anti-small mother against decapentaplegic (Smad)2/3 (Abcam, ab202445, diluted to 1:1000), phosphorylated (p)-Smad2/3 (Abcam, ab280888, diluted to 1:500), Smad7 (Abcam, ab216428, diluted to 1:500) and NAD(P)H oxidase 4 (NOX4) (Abcam, ab133303, diluted to 1:2000). After being washed with 1 × PBS solution (Sangon, Shanghai, China) three times, the membranes were incubated with HRP-labelled goat anti-rabbit IgG secondary antibodies (Abcam, ab205718, diluted to 1:10,000). Immunoreactivity was determined with enhanced chemiluminescence (ECL) reagent (Thermo Fisher, US). A gel imaging system (BIO-RAD Gel Doc XR + , US) and software (BIO-RAD Image Lab Software, Version 5.1 and SPSS 20.0) were used for imaging and statistical analysis. GAPDH was used as an internal control to ensure equal protein loading.

### Fluorescein-conjugated phalloidin staining

The podocyte cytoskeletal remodelling with different treatments in this study were detected by fluorescein-conjugated phalloidin staining using test kits (APExBIO, Beijing, China) according to the manufacturer's instructions.

### Animal experiment

The Wistar rats purchased from Beijing Vital River Laboratory Animal Technology Co. Ltd. were divided into two groups randomly. The control group rats were with free access to water and normal diet. The modelled DN rats were supplied with high-fat-sugar diet (10% lard, 10% egg yolk powder, 5% sucrose), free access to water, and intraperitoneal injected with streptozotocin (70 mg/kg) six week later to induced DN. The DN rats were divided into two groups randomly. The klotho intervention rats were intraperitoneal injected with klotho proteins (0.02 mg/kg) for 7 d consecutively. The control and DN group were intraperitoneal injected with normal saline. The health status was monitored by a specific veterinarian. All animals were euthanized and sacrificed by cervical dislocation after treatment, and the kidney tissues were removed. All experiments with animals were approved by the Institutional Animal Care and Use Committee of Soochow University.

### Immunohistochemistry

The kidney tissues were embedded in paraffin and cut into 4-μm in thickness. After dewaxed and hydrated, the sections were washed with distilled water containing 3% hydrogen peroxidase to reduce endogenous oxidase activity. Then the tissue sections were incubated with anti-SRGAP2a antibody (Abcam, ab121977), anti-nephrin (Abcam, ab216692), anti-synaptopodin (Abcam, ab224491) for 2 h at room temperature, and subsequently, incubated with goat-anti-rabbit antibody at room temperature for 40 min. The degree of staining was determined by developing with diaminobenzidine (DAB) chromogen (Bio-Rad, Inc., CA, USA).

### Intracellular ROS generation analysis

We measured intracellular ROS generation with the fluorescent probe DCFH-DA, which is hydrolysed and generates nonfluorescent DCFH after passing through the cell membrane. The intracellular ROS oxidizes DCFH to produce fluorescent DCF. The level of DCF fluorescence intensity indicates the level of intracellular ROS.

Podocyte samples were treated as described above for qRT-PCR or western blotting, washed with cold 1 × PBS solution once, resuspended in serum-free DMEM and incubated with DCFH-DA (10 μM)) for 30 min at 37 °C. The samples were mixed every 3–5 min to ensure good contact between the probes and podocytes. After that, the podocytes were washed with serum-free DMEM three times and resuspended in 1 × PBS solution. Finally, the podocyte samples were analysed by flow cytometry (Life Attune NxT, US). FlowJo 10 software was used for data analysis.

### Statistical analysis

Sigma Plot 12.0 and SPSS 20.0 were used for statistical analysis. All data are presented as the means ± SD. Independent group comparisons were performed using Student’s *t*-test or one-way ANOVA with Bonferroni’s post hoc test. A value of *P* < *0.05* was considered statistically significant.

## Results

### The culture of rat podocytes

**Fig. 1:**
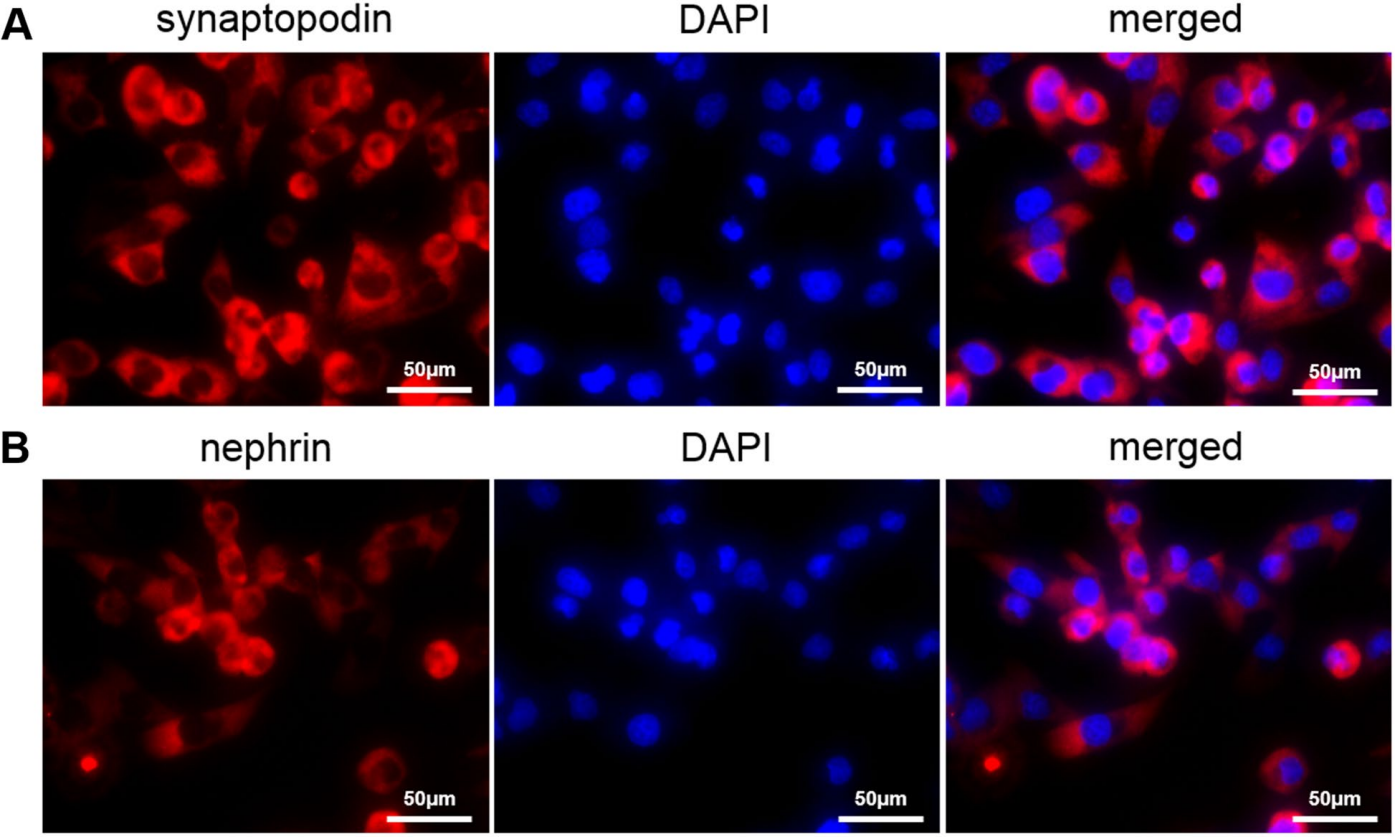
The immunofluorescence staining to label synaptopodin **A** and nephrin **B** of the cultured differentiated rat podocytes

Podocytes are important targets in various kidney diseases. In this study, we performed all experiments in cultured differentiated rat podocytes. To harvest podocytes that were suitable for further study, we performed immunofluorescence labelling for nephrin (Abcam, UK) and synaptopodin (Abcam, UK), which are specific markers for differentiated podocytes (Fig. 1). The immunofluorescence results indicated that the cultured differentiated rat podocytes were pure enough for further study.

### SRGAP2a involved in the TGF-β1 and ROS signalling pathway

**Fig. 2:**
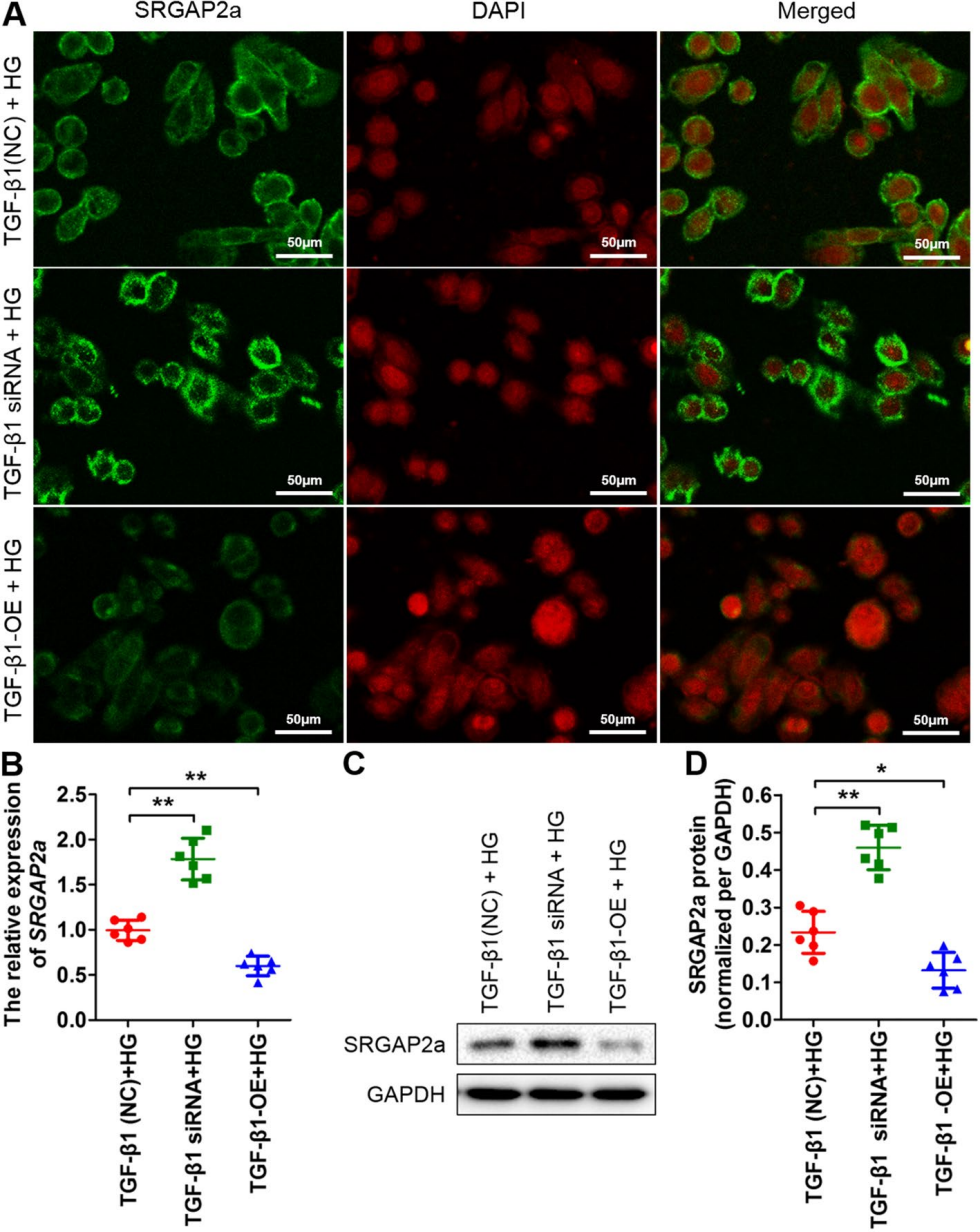
SRGAP2a is involved in the TGF-β1 signalling pathways in rat podocytes. **A** The immunofluorescence staining of SRGAP2a in TGF-β1 interference and TGF-β1 overexpression conditions with HG treatments. **B** The qRT-PCR results showed that the expression of SRGAP2a in silencing TGF-β1 + HG treatment and in TGF-β1-OE + HG treatment. **C** The western blot results showed the SRGAP2a levels in silencing TGF-β1 + HG treatment and in TGF-β1-OE + HG treatment. **D** The quantitative analysis of the SRGAP2a protein levels in **C**) All data were mean ± SD, (*n* = 6); *p < *0.05*, **p < *0.01*. Bars = 50 μm in **A**

**Fig. 3:**
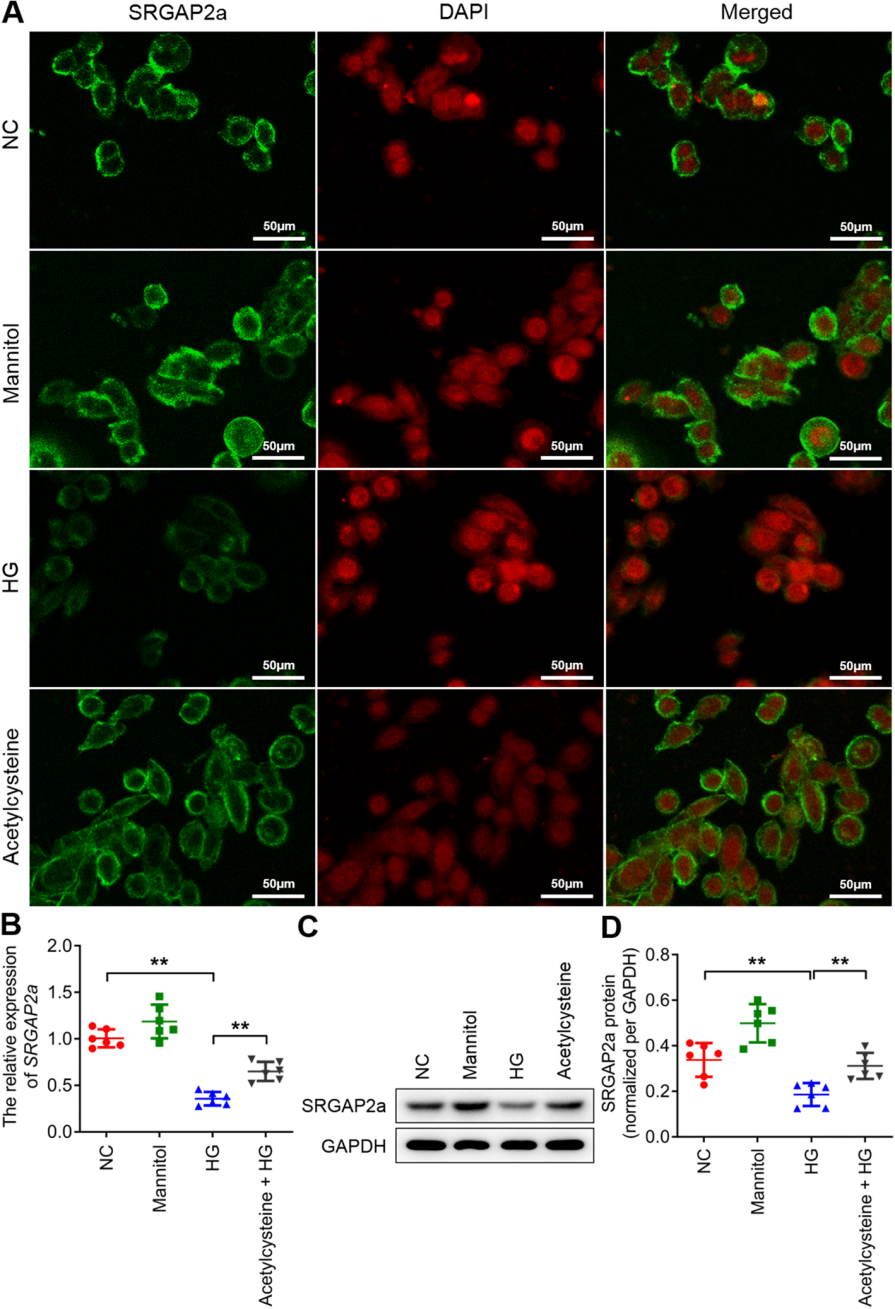
SRGAP2a is involved in the ROS signalling pathways in rat podocytes. **A** The immunofluorescence staining of SRGAP2a after inhibiting ROS generation. **B** The qRT-PCR results showed that the expression of *SRGAP2a* after inhibiting ROS generation. **C** The western blot results showed that the SRGAP2a level increased after inhibiting ROS generation. **D** The quantitative analysis of the SRGAP2a protein levels in **C**. All data were mean ± SD, (*n* = 6); *p < *0.05*, **p < *0.01*. Bars = 50 μm in **A**. NC, negative controls without additional treatment

The expression of SRGAP2a is downregulated in cultured podocytes treated with TGF-β1, and TGF-β1 may induce the production of ROS by increasing the expression of NOX4 in human pulmonary artery smooth muscle cells [7, 34]. Thus, we focused on the function of SRGAP2a in the TGF-β1 and ROS signalling pathways. According to the results from qRT-PCR and western blotting, TGF-β1 expressions were upregulated with HG treatment (**p < *0.01*) (Supplemental Fig. 1). We performed siRNA-mediated silencing of TGF-β1 and TGF-β1 overexpression (Fig. 2; Supplemental Fig. 2). The results from immunofluorescence staining showed that SRGAP2a signals increased in TGF-β1 siRNA group, but decreased in TGF-β1-OE group, compared to the control without TGF-β1 silencing under HG conditions (Fig. 2A). The results from qRT-PCR were in consistent with the immunofluorescence staining experiment that the mRNA expression of SRGAP2a showed significantly increased in TGF-β1 siRNA group, and decreased significantly in TGF-β1-OE group, compared to the controls (**p < *0.01*) (Fig. 2B). The western blot analysis further verified the above results (Fig. 2C and D). The SRGAP2a signals from immunofluorescence staining in the presence of HG treatment was downregulated compared to the NC groups (Fig. 3A). When ROS inhibitors were added to the HG solutions, the SRGAP2a signals were obviously enhanced (Fig. 3A). The qRT-PCR and western blot results were consistent with the immunofluorescence staining findings (Fig. 3B-D). These results indicate that SRGAP2a is involved in TGF-β1 and the ROS signalling pathway in rat podocytes.

### Klotho increased SRGAP2a levels in HG-induced rat podocytes

**Fig. 4:**
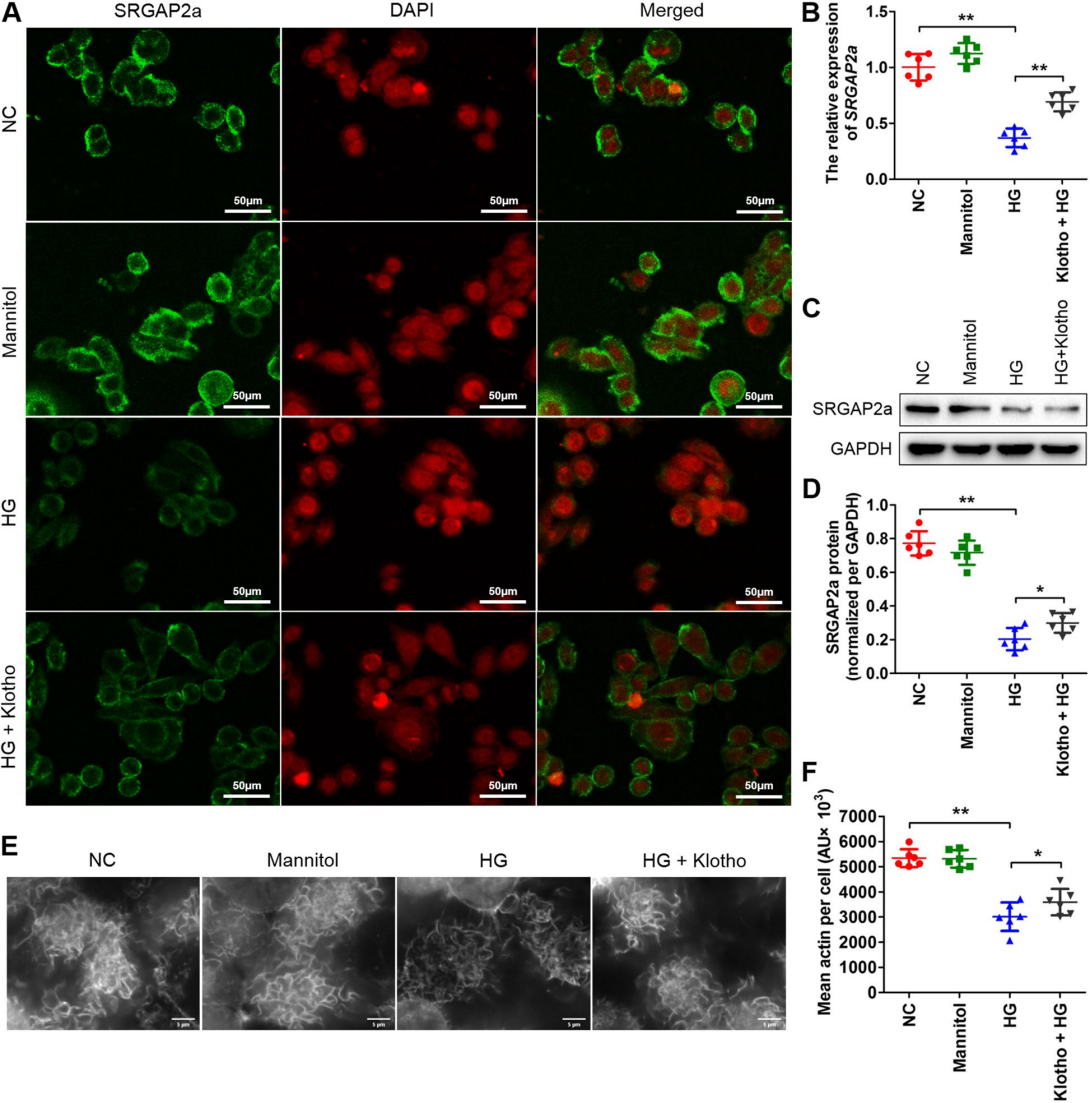
The SRGAP2a expressions and actin cytoskeleton of podocytes following HG and HG plus klotho treatment. **A** The immunofluorescence staining of SRGAP2a with HG and HG plus klotho treatment. **B** The qRT-PCR results showing SRGAP2a mRNA expressions with HG and HG plus klotho treatment. **C** The western blotting results showing SRGAP2a protein expressions with HG and HG plus klotho treatment. **D** The quantitative analysis of the SRGAP2a protein levels in **C**. **E** The fluorescein-conjugated phalloidin staining of podocytes with HG and HG plus klotho treatment. **F** The quantitative analysis of the mean actin content per cell in **E**. All data were mean ± SD, (*n* = 6); *p < *0.05*, **p < *0.01*. Bars = 50 μm in **A**, 5 μm in **E**. NC, negative controls without additional treatment

To examine the effects of klotho on SRGAP2a, we performed immunofluorescence staining, qRT-PCR and western blot analysis of rat podocytes. The immunofluorescence staining results showed that the SRGAP2a signals decreased when the cells were treated with HG compared to the negative controls or mannitol groups (Fig. 4A) but increased obviously after the addition of soluble klotho protein to the HG solutions (Fig. 4A). The qRT-PCR results were in consistent with the immunofluorescence staining that the soluble klotho addition promoted the mRNA expressions of SRGAP2a sinificantly (**p < *0.01*) (Fig. 4B). Western blot analysis showed that the SRGAP2a protein level decreased significantly with HG treatment and increased significantly after klotho supplementation in HG solutions (**p < *0.01*) (Fig. 4C and D), which confirmed the qRT-PCR results. Taken together, these results suggested that klotho could efficiently induce SRGAP2a expression under HG conditions in rat podocytes. In addition, podocyte cytoskeletons staining by fluorescein-conjugated phalloidin showed that high concentration of glucose significantly disrupted podocyte cytoskeleton, but klotho addition largely rescued the disruption of F-actin stress fiber in podocytes caused by treatment with high concentration of glucose. (*p < *0.05*, **p < *0.01*) (Fig. 4E and F).

### Klotho regulated the expression of key factors in the TGF-β1/Smad and ROS/NOX4 signalling pathways

**Fig. 5:**
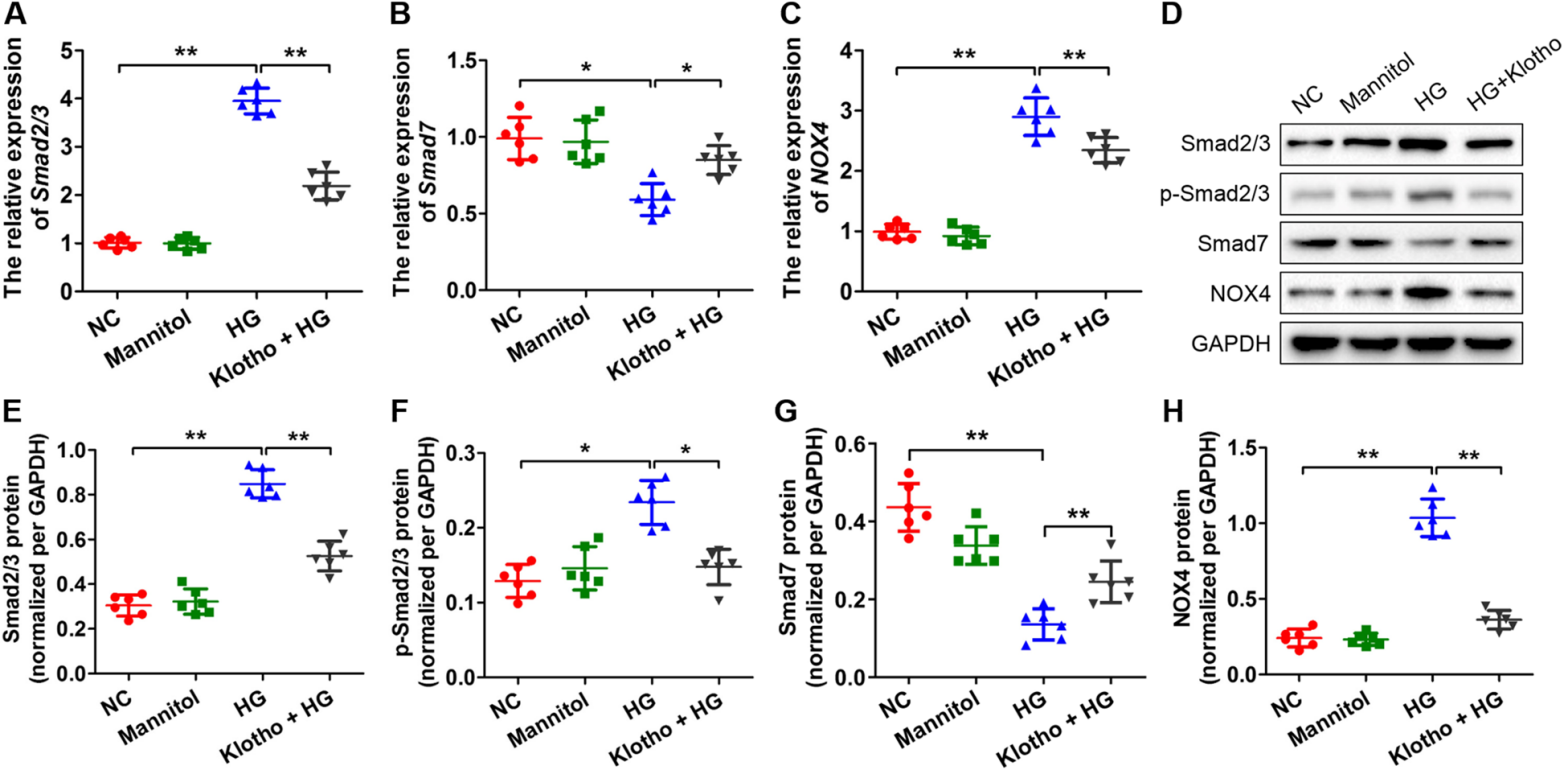
Klotho regulated the expression of Smad2/3*,* p-Smad2/3, Smad7, and NOX4. **A-C** The qRT-PCR results showed the expression of Smad2/3, Smad7, and NOX4 with HG and HG plus klotho treatments compared with the NC groups. **D** The western blot results showed that the protein levels of Smad2/3, p-Smad2/3, Smad7, and NOX4 changed with HG and HG plus klotho treatments compared with the NC groups. **E–H** The quantitative analysis of the results from **D**. All data were mean ± SD, (*n* = 6); *p < *0.05*, **p < *0.01*

**Fig. 6:**
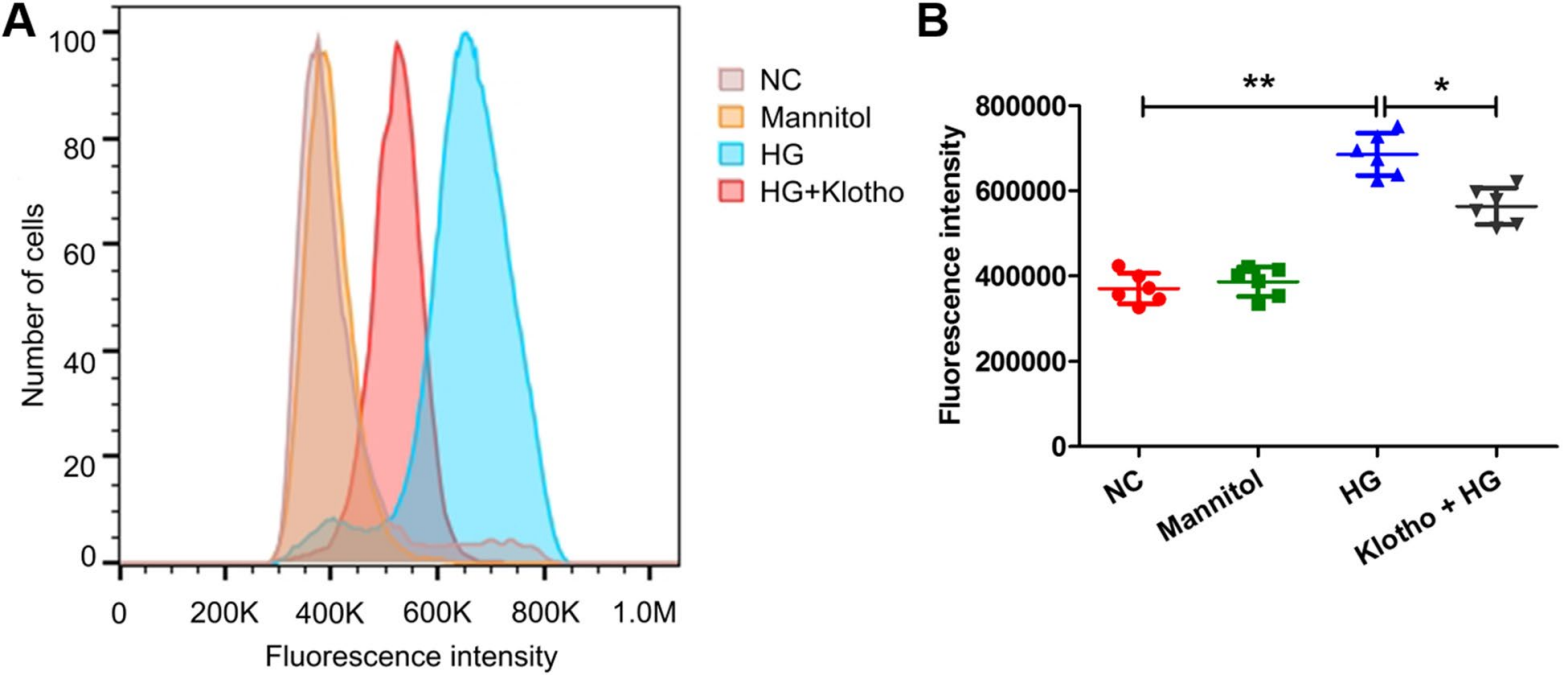
ROS generation with HG and HG plus klotho treatments. **A** Flow cytometry analysis showed that ROS generation in the HG and HG plus klotho treatments changed compared with that in the NC groups. **B** Statistical analysis of ROS generation, as determined by flow cytometry analysis. All data were mean ± SD, (*n* = 6); *p < *0.05*, **p < *0.01*

To determine whether klotho prevents kidney injury by regulating TGF-β1 and ROS signalling in podocytes, we measured the expression of some key factors in the pathways. We quantified the expression of Smad2/3, Smad7 and NOX4 by qRT-PCR, which showed that the mRNA levels of Smad2/3 and NOX4 significantly increased in response to HG treatments compared to the NC groups but decreased significantly after the addition of klotho to the HG solutions (*p < *0.05*, **p < *0.01*) (Fig. 5A and C). Conversely, the transcript levels of Smad7 decreased significantly after HG treatment but slightly increased after klotho intervention (*p < *0.05*) (Fig. 5B). The protein levels of Smad2/3, Smad7 and NOX4 measured by western blotting were consistent with the results from qRT-PCR (*p < *0.05*, **p < *0.01*) (Fig. 5D, E, G and H). The protein level of p-Smad2/3 was significantly increased with HG treatment but decreased by klotho intervention (*p < *0.05*) (Fig. 5D and F). In addition, we measured ROS generation by flow cytometric analysis, which showed that ROS generation was increased by approximately 1.7 times with HG treatment (**p < *0.01*) (Fig. 6). However, the ROS levels significantly decreased after klotho intervention (*p < *0.05*) (Fig. 6). These results demonstrated that klotho could regulate the expression of key factors in the TGF-β1/Smad and ROS/NOX4 pathways in podocytes.

### Klotho protects kidney injury in diabetic rats

**Fig. 7:**
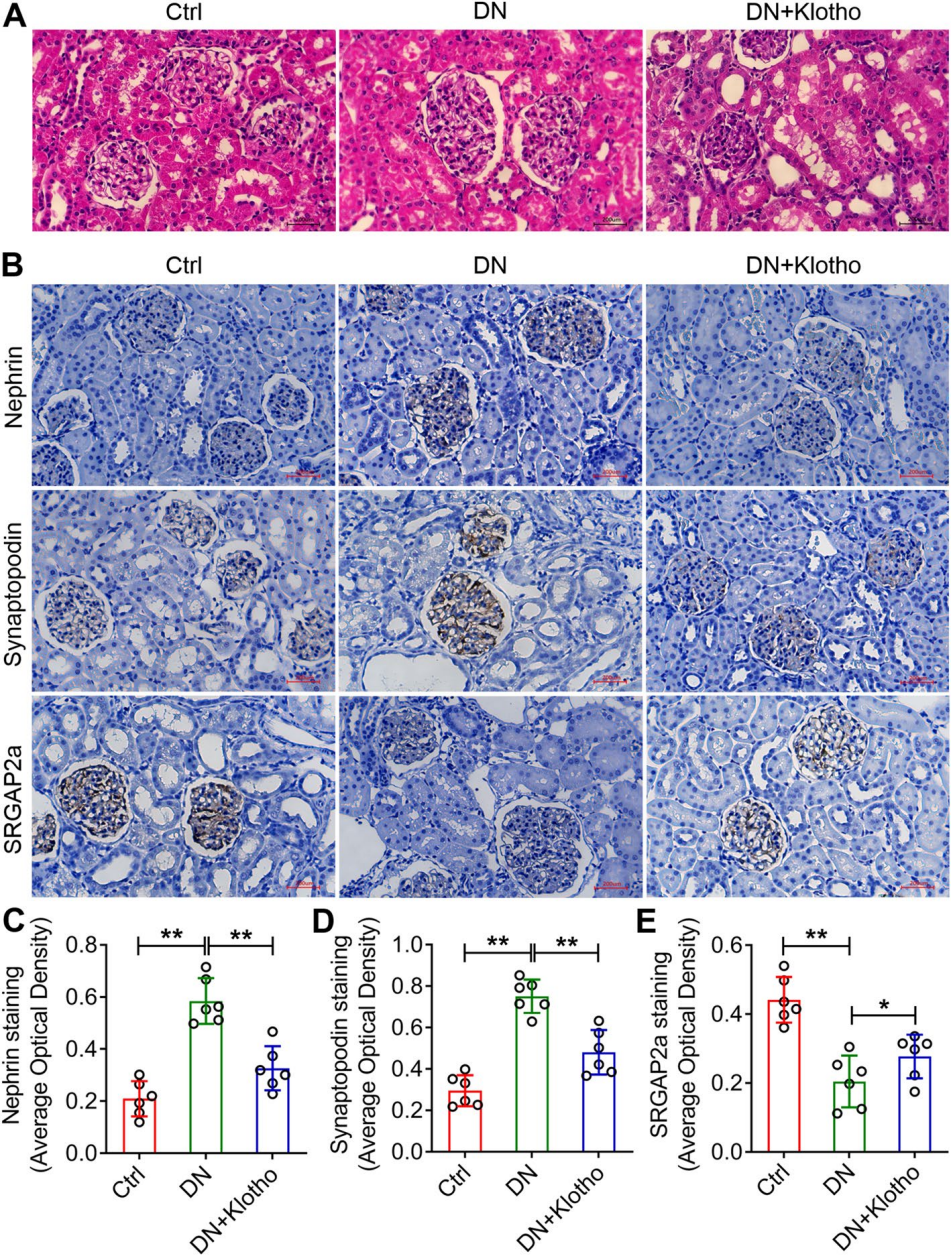
The HE staining **A** and Nephrin, Synaptopodin and SRGAP2a immunochemistry **B** of the kidneys from the modelled DN and DN plus klotho rats. Quantitative analysis of the average optical density of Nephrin **C**, Synaptopodin **D** and SRGAP2a **E** by immunohistochemistry. Bars = 200 μm. All data were mean ± SD, (*n* = 6); *p < *0.05*, **p < *0.01*

To figure out the effect of klotho in diabetic podocyte injury, we established the diabetic rats. The blood glucose, creatinine, urea level, total cholesterol level and triglyceride content were detected (Table 1). The results from haematoxylin–eosin (HE) staining showed that rat kidney glomeruli were hypertrophic, mesangial matrix increased and erythrocyte stasis was visible in DN rats (Fig. 7A). Obvious vacuoles appeared in the cytoplasm of kidney tubular epithelial cells (Fig. 7A). These pathological changes in DN kidney were rescued obviously by klotho supplement (Fig. 7A). Moreover, the SRGAP2a and podocyte makers of nephrin and synaptopodin protein expressions were decreased in DN rats but increased visibly after klotho administration from immunochemistry (Fig. 7B).

**Table 1:** Blood glucose, creatinine, urea level, total cholesterol level and triglyceride content of each group

Group	BG (mmol/L)	CREA (μmol/L)	UREA (mmol/L)	TC(mmol/L)	TG(mmol/L)
Ctrl	5.74 ± 0.15	42.41 ± 10.22	39.12 ± 2.28	1.26 ± 0.18	0.58 ± 0.12
CN	22.81 ± 0.86**	75.90 ± 16.07*	58.04 ± 2.35*	2.32 ± 0.19*	1.33 ± 0.23**
CN + Klotho	17.06 ± 0.52*	63.58 ± 9.19*	45.23 ± 3.19*	1.64 ± 0.21*	0.74 ± 0.30**

Values are means ± SE (*n* = 10). CN group vs control group, CN + Klotho group vs CN group. ** *P* < *0.01*

## Discussion

DN is a common complication of diabetes mellitus, the major cause of kidney failure and the leading cause of CKD [8, 35]. Podocyte injury, which is clinically indicated by progressive decline in the eGFR and increased proteinuria, plays a critical role in the pathogenesis of DN [5–8]. The specific function of podocytes is strongly dependent on the actin cytoskeletons of the interdigitating foot process between neighbouring podocytes [5, 7]. SRGAP2a was strongly suggested to protect podocyte function and structure [7]. In this study, we found that the expression of SRGAP2a was significantly decreased in cultured podocytes under HG conditions. And the DN rats also revealed downregulation of SRGAP2a expression. These results were consistent with the investigation showing that the SRGAP2a level was significantly decreased in DN patients [7]. These findings indicated that the expression of SRGAP2a might be essential for proper podocyte activity.

TGF-β signalling regulates a series of biological processes and is indispensable in animal development [36]. Smad transcription factors occupy the core of this pathway [37]. According to this study, TGF-β1 silencing stimulated SRGAP2a expression under HG conditions in podocytes. In addition, previous studies revealed that TGF-β1 significantly reduced the podocyte level of SRGAP2a [7]. In summary, the molecular functions of SRGAP2a seemed to be tightly associated with TGF-β1/Smad signalling. ROS inhibition resulted in the upregulation of podocyte SRGAP2a expression which indicated that SRGAP2a might be involved in ROS signalling. Previous research indicated that HG-induced ROS production increased TGF-β1 level in DN [32]. Overall, we suggest that SRGAP2a plays protective roles against kidney diseases via the TGF-β1/Smad/ROS signalling axis. Increased ROS generation (oxidative stress) is consequently associated with the hyperglycaemia underlying DN [38–40]. Thus, we hypothesised that reducing oxidative stress might facilitate SRGAP2a expression to prevent podocyte injury in DN, since in diabetic mice, reducing oxidative stress efficiently improved kidney structure and function [41].

On the one hand, the serum klotho protein level is one of the early markers of kidney diseases, as the level declines at the early stage of CKD and continuously decreases with the progression of CKD [24]. On the other hand, klotho protein is considered to be a novel therapeutic target [22, 24, 42]. In this study, we found that the expression of SRGAP2a significantly increased after the addition of klotho to HG solutions compared with the expression of SRGAP2a in HG-treated cultured podocytes. This might indicate that klotho protein actually stimulated SRGAP2a expression in podocytes to protect against injury. In addition, the expression of key factors in the TGF-β1/Smad/ROS signalling pathway changed after klotho administration. The expression of NOX4 and Smad2/3 was greatly enhanced with HG treatment but significantly downregulated when klotho was added to the HG treatment. However, the expression of Smad7, an inhibitory Smad serving as a competitor for receptor-regulated Smad proteins such as Smad2/3, was contrary to the results of Smad2/3 and NOX4. The protein level of p-Smad2/3 showed the same trend as that of Smad2/3 and NOX4 in the presence of HG and exogenous klotho. These results suggested that klotho could regulate the expression of key factors in TGF-β1/Smad/ROS signalling that are strongly associated with SRGAP2a in podocytes. NOX4 is the major source of ROS in the kidney [38–40]. NOX4-derived ROS mediate TGF-β1/Smad signalling in many disease processes. In this study, ROS generation was analysed by flow cytometry and was enhanced under HG conditions but reduced significantly after klotho administration. In summary, exogenous klotho reduced ROS generation, which might benefit the expression of SRGAP2a to prevent podocyte injury in DN. The TGF-β1/Smad/ROS signalling pathway may regulate actin activity in kidney myofibroblast activation, human pulmonary artery smooth muscle cells and kidney interstitial fibrosis [34, 43, 44]. In this study, klotho remodelled the actin fibers in DN rats. Thus, klotho may prevent podocyte dysfunction by regulating the expression of SRGAP2a and key factors of the associated regulatory pathway.

## Conclusion

This study mimicked the medical treatment of DN patients with klotho protein. Our findings indicated that soluble klotho could interfere the expressions of SRGAP2a and key molecules in TGF-β1/Smad/ROS signalling in cultured podocytes and maintaining the podocyte cytoskeleton under HG conditions, and protecting kidney injury in DN rats. Further studies are necessary to precisely understand the role of klotho in protecting podocytes from DN. This research provides theoretical support for klotho protein as a novel treatment for DN patients.

## Supplementary Information

**Additional file 1: Supplemental Figure 1.** The mRNA (A) and protein (B and C) expressions of TGF-β1 under HG condition. All data were mean ± SD, (n = 6); *p < 0.05, **p < 0.01. **Supplemental Figure 2**. The mRNA expression of TGF-β1 inthe siRNA-mediated TGF-β1 silencing (A) and overexpression (B) podocytes. Alldata were mean ± SD, (n = 6); *p < 0.05, **p < 0.01.

**Additional file 2: Supplemental Table 1.** Sequence information for primers used in qRT-PCR.

## Data Availability

The datasets used and/or analyzed during the current study are available from the corresponding author on reasonable request.

## Abbreviations

DN: Diabetic nephropathy
SRGAP2a: Slit-Robo GTP activating protein 2a
ROS: Reactive oxygen species; Smad, small mother against decapentaplegic
p-Smad: Phosphorylated-Smad
NOX4: NAD(P)H oxidase 4
HG: High glucose
HE: Haematoxylin-eosin
CKD: Chronic kidney disease
GBM: Glomerular basement membrane
ADAM: A disintegrin and metalloproteinase
TGF-β1: Transforming growth factor β1
eGFR: Estimated glomerular filtration rate
DMEM: Dulbecco's modified Eagle's medium
BCA: Bicinchoninic acid
SDS-PAGE: Sodium dodecyl sulfate–polyacrylamide gel electrophoresis
DAB: Diaminobenzidine
