# Supplementary material for: Impact of klotho on the expression of SRGAP2a in podocytes in diabetic nephropathy
Source: BMC Nephrol. 2022 Apr 18;23:151. doi: 10.1186/s12882-022-02765-z (PMC9014571; doi:10.1186/s12882-022-02765-z)
Supplement: Supplementary file 1 — Additional file 1: Supplemental Figure 1. The mRNA (A) and protein (B and C) expressions of TGF-β1 under HG condition. All data were mean ± SD, (n = 6); *p < 0.05, **p < 0.01. Supplemental Figure 2. The mRNA expression of TGF-β1 inthe siRNA-mediated TGF-β1 silencing (A) and overexpression (B) podocytes. Alldata were mean ± SD, (n = 6); *p < 0.05, **p < 0.01. [file 12882_2022_2765_MOESM1_ESM.docx]

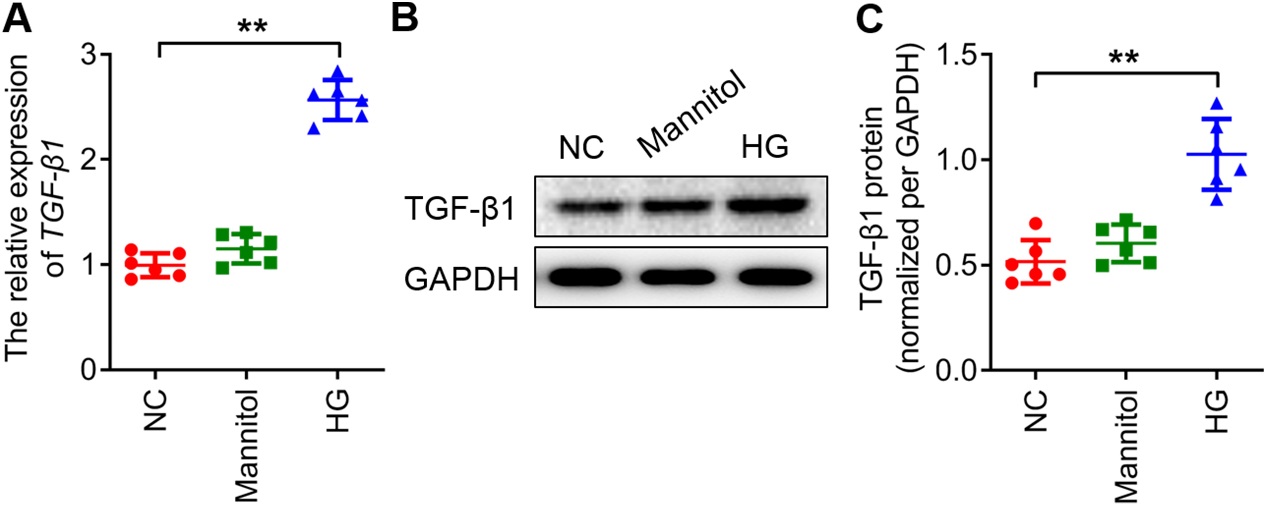


Supplemental Figure 1. The mRNA (A) and protein (B and C) expressions of TGF-β1 under HG condition. All data were mean ± SD, (n = 6); **p* < 0.05, ***p* < 0.01.


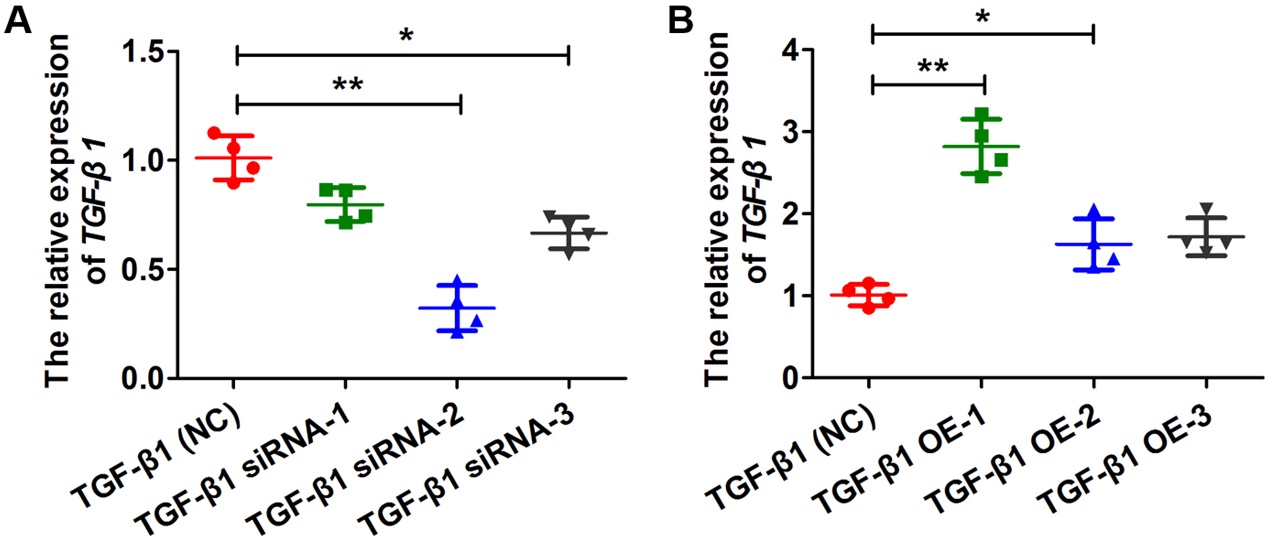


Supplemental Figure 2. The mRNA expression of TGF-β1 in the siRNA-mediated TGF-β1 silencing (A) and overexpression (B) podocytes. All data were mean ± SD, (n = 6); * *p* < 0.05, ** *p* < 0.01.
