# Supplementary material for: Impact of klotho on the expression of SRGAP2a in podocytes in diabetic nephropathy
Source: BMC Nephrol. 2022 Apr 18;23:151. doi: 10.1186/s12882-022-02765-z (PMC9014571; doi:10.1186/s12882-022-02765-z)
Supplement: Supplementary file 2 — Additional file 2: Supplemental Table 1. Sequence information for primers used in qRT-PCR. [file 12882_2022_2765_MOESM2_ESM.docx]

| Table 1. Sequence information for primers used in qRT-PCR. | | |
| --- | --- | --- |
| **Gene ID** | **Forwad primer（5’-3’）** | **Reverse primer（5’-3’）** |
| 81822 | CATCACTGCCACCCAGAAGACTG | ATGCCAGTGAGCTTCCCGTTCAG |
| 14270 | TTCGGAGGAGCCGTGGTCCGCG | GTCTGCGCACACCGCCGCCTCAC |
| 85431 | GTATGTTTAGTTCCAATCGTCAG | ACATTATGGGTACTGAAGCAA |
| 29357 | GGATTGAGAGTCAAGATTGGGCA | ATTGGCTTCTCAAGATACCT |
| 81516 | ATCTGAAGGCGCTCACGCACT | AGATCCGGCCACCTGAACACTT |
| 59086 | CTGGACACACAGTACAGCAAGGTCC | ATCATGTTGGACAACTGCTCCACCT |
